# Photosensitizer-Trapped Gold Nanocluster for Dual Light-Responsive Phototherapy

**DOI:** 10.3390/biomedicines8110521

**Published:** 2020-11-20

**Authors:** Junho Byun, Dongyoon Kim, Jaehyun Choi, Gayong Shim, Yu-Kyoung Oh

**Affiliations:** 1 College of Pharmacy and Research Institute of Pharmaceutical Sciences, Seoul National University, Seoul 08826, Korea; junho_byun@snu.ac.kr (J.B.); mmamic@snu.ac.kr (D.K.); cjhopen@snu.ac.kr (J.C.); 2 School of Systems Biomedical Science, Soongsil University, Seoul 06978, Korea

**Keywords:** photodynamic therapy, photothermal therapy, dual light-responsive nanomaterials

## Abstract

Photoresponsive nanomaterials have recently received great attention in the field of cancer therapy. Here, we report a photosensitizer-trapped gold nanocluster that can facilitate dual light-responsive cancer therapy. We utilized methylene blue (MB) as a model photosensitizer, gold nanocluster as a model photothermal agent, and a polymerized DNA as the backbone of the nanocluster. We synthesized MB-intercalated gold DNA nanocluster (GMDN) via reduction and clustering of gold ions on a template consisting of MB-intercalated long DNA. Upon GMDN treatment, cancer cells revealed clear cellular uptake of MB and gold clusters; following dual light irradiation (660 nm/808 nm), the cells showed reactive oxygen species generation and increased temperature. Significantly higher cancer cell death was observed in cells treated with GMDN and dual irradiation compared with non-irradiated or single light-irradiated cells. Mice systemically injected with GMDN showed enhanced tumor accumulation compared to that of free MB and exhibited increased temperature upon near infrared irradiation of the tumor site. Tumor growth was almost completely inhibited in GMDN-treated tumor-bearing mice after dual light irradiation, and the survival rate of this group was 100% over more than 60 days. These findings suggest that GMDN could potentially function as an effective phototherapeutic for the treatment of cancer disease.

## 1. Introduction

External stimulus-responsive systems are being proposed as a new alternative modality in the field of cancer-targeted therapy [1,2]. Photo-responsive agents have been particularly studied to achieve complete tumor ablation through photothermal [3,4] or photodynamic [2,5] effects, which generate heat or reactive oxygen species (ROS), respectively, upon light irradiation. Photothermal treatment using near-infrared (NIR) light has various advantages over conventional anti-cancer therapy [3,6]. For example, phototherapy facilitates minimally invasive treatment by irradiating light to the diseased area, and thereby prevents systemic toxicity. Moreover, photothermal therapy-mediated hyperthermic cell death provides a molecular switch that can trigger cell death without the limitations of chemotherapy, such as severe side effects and drug resistance.

However, there are a few remaining concerns surrounding the use of photoresponsive materials as a new therapeutic. For example, in vivo fate of photoresponsive materials is important issue because it can produce effective therapeutic effects through light irradiation to the target area under optimal conditions. Modification of the photo-responsive materials—gold [7], carbon [8], and polymers [9]—has been intensively studied as a means to track the in vivo behavior of nanomaterials for theranostic purposes. However, the above-listed photoresponsive materials suffer from the quenching of fluorescence when the fluorescent probe interacts with the hydrophobic surface of the nanoparticles, and it is a cumbersome process to insert a spacer or a cleavable linker molecule to prevent this phenomenon. In addition, since tumor recurrence is often observed in an irradiated lesion after phototherapy, the phototherapeutic efficacy needs to be improved to ensure complete eradication of tumor tissues [10]. In terms of clinical development, some photoresponsive materials have limitations due to toxicity, safety issues, and/or unverified excretion profiles [11,12]. 

**Figure 1:**
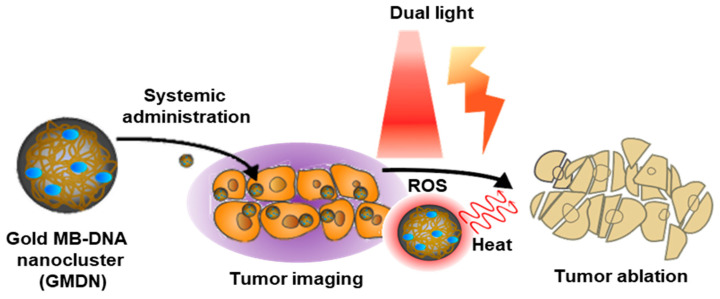
Schematic illustration. For synergistic dual phototherapy, methylene blue (MB) was trapped in gold DNA nanoclusters to form MB-trapped gold DNA nanoclusters (GMDN). After systemic administration, tumor accumulation of GMDN was monitored by molecular imaging. Then, dual light irradiation (660 nm and 808 nm) was applied to induce photodynamic and photothermal therapy for tumor ablation.

**Figure 2:**
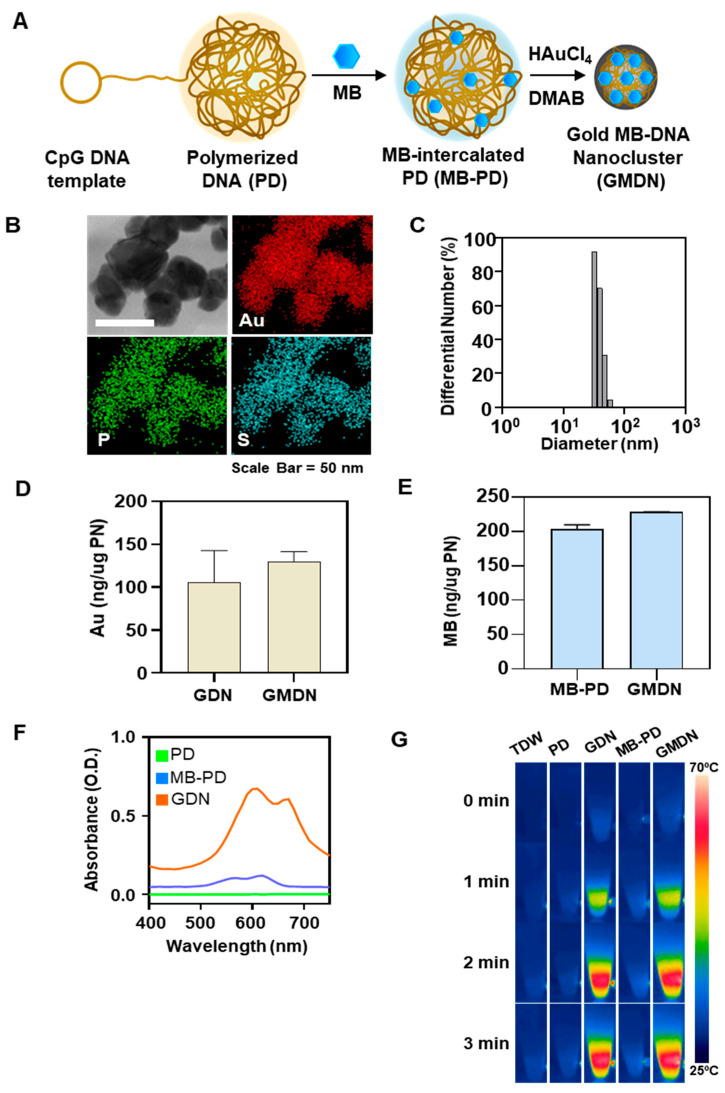
Characterization of nanoclusters. (**A**) Schematic illustrations of GMDN preparation. (**B**) Elemental mapping images obtained by energy dispersive X-ray spectroscopy-scanning transmission electron microscopy (EDS-STEM) for gold, phosphorous, and sulfur. Scale bar: 50 nm. (**C**) Size distribution of GMDN. (**D**) Au contents of gold DNA nanoclusters (GDN) and GMDN were evaluated by inductively coupled plasma mass spectrometry (ICP-MS). (**E**) Loading amounts of methylene blue (MB) in MB-intercalated polymerized CpG DNA (MB-PD) and GMDN were measured by fluorescence spectrometry. (**F**) Absorbances of polymerized CpG DNA (PD), MB-PD, and GMDN were assessed by UV/Vis spectrometry. (**G**) Photoresponsive properties of various groups were observed by temperature monitoring during NIR irradiation.

In this study, we designed a dual light-responsive DNA-based nanocluster to achieve an effective image-guided dual phototherapy that is capable of dual photothermal and photodynamic therapy for theranostic purposes (Figure 1). We selected methylene blue (MB) as a model photosensitizer and gold (iii) ion as the seed material for the photothermal nanocluster. We synthesized a polymerized DNA fiber via rolling circle amplification (RCA); this fiber serves multiple functions, acting as a safe and biodegradable backbone, a delivery carrier of MB, and a template for the gold nanocluster. MB is a well-known photosensitizer and clinically used for the treatment of methemoglobinemia with US Food and Drug Administration approval (Provayblue^®^) [13]. Several studies reported the applications of MB for photodynamic anticancer therapy [14,15,16]. Equipped with the DNA intercalating ability, MB was loaded in DNA nanostructures [14,15]. With the light-to-heat conversion feature, gold in nanomaterials was studied for photothermal therapy [17,18,19]. Photothermal gold nanomaterials have been investigated in various shapes of nanosphere, nanoshell, nanorod, and nanocluster. The application of gold nanoshells for phototherapy of prostate tumors is in a clinical trial [20]. Taken together, the clinical reports of MB and gold nanomaterials suggest the potential of MB and gold nanocluster for translational studies. MB-trapped gold DNA nanoclusters (GMDN) were generated by reducing gold ions with MB-intercalated long DNA as a cluster template (Figure 2A). GMDN yielded acceptable tumor ablation efficacy due to the response to dual light (660 nm for MB, 808 nm for gold) irradiation. Moreover, the in vivo behavior of GMDN can be traced by monitoring the entrapped MB, which has long wavelength-excitable fluorescence. Here, we report that GMDN can exert phototherapeutic effects via combined photodynamic and photothermal therapy. Moreover, GMDNs allow image-guided phototherapy, and thus show potential as a theranostic nanoplatform.

## 2. Experimental Section

### 2.1. Preparation of GMDN

GMDNs were prepared by clustering, which occurred when Au^3+^ ions were reduced with MB-intercalated DNA. An oligo primer (5′-TATATACTAGTCAGATATTACT-3′) and a linear DNA sequence (5′-ATCTGACTAGTATATAAACGTCAGGAACGTCATGGAAACGTCAGGAACGTCATGGAAGTAAT-3′) were used to produce polymerized CpG DNA (PD) as previously reported [16]. The linear DNA template was annealed with the primer (Macrogen Inc., Daejeon, Korea) in hybridization buffer (10 mM Tris–HCl, 1 mM EDTA, 100 mM NaCl, pH 8.0), and then incubated with T4 DNA ligase (125 units/mL; Thermo Scientific, Waltham, MA, USA); this ligated any nicks in the hybridized DNA complex to generate a circular template. Inactivation of T4 DNA ligase was performed at 70 °C for 5 min. This was followed by DNA amplification, which was performed using phi29 DNA polymerase (100 units/mL; Thermo Scientific) and 2 mM of dNTPs (ELPIS Biotech, Inc., Daejeon, Korea) for 24 h at 30 °C. Free dNTPs were removed by centrifugation at 11,000× *g* for 3 min, and the resulting PD was resuspended in triple-distilled water (TDW). MB was intercalated to PD by incubating 25 μL of PD (10 μg) with 75 μL of MB (100 μM) for 5 min. The resulting MB-intercalated PD (MB-PD) was purified by centrifugation at 11,000× *g* for 3 min and resuspended in 180 μL of TDW. To prepare GMDN, 10 μL of HAuCl_4_∙3H_2_O (50 mM; Sigma-Aldrich, St. Louis, MO, USA) and 10 μL of dimethylamine borane (5 mM, DMAB; Sigma-Aldrich) were added to 180 μL of MB-PD with vigorous mixing. Gold DNA nanoclusters (GDN) were prepared under the same conditions but without loading of MB. For further experiments, various groups were prepared in aqueous 5% glucose solution.

### 2.2. Characterization of GMDN

The physicochemical properties of various nanoclusters were evaluated in terms of their size distribution, morphology, electron mapping, absorbance, cargo-loading efficiency, and photo-responsivity. To evaluate morphology and perform electronic mapping of phosphorus and sulfur, nanoparticles were observed by energy dispersive X-ray spectroscopy-scanning transmission electron microscopy (EDS-STEM) using a JEM-2100 F transmission electron microscope (TEM; JEOL Ltd., Tokyo, Japan). To measure the particle size distribution, dynamic light scattering was applied with an ELSZ-1000 instrument (Otsuka Electronics Co., Osaka, Japan). The zeta potential was measured by laser Doppler micro electrophoresis at an angle of 22° with an ELSZ-1000 instrument (Otsuka Electronics). The UV/Vis spectra of RCA products were obtained using a SpectraMAX M5 (Molecular Devices Corp., Sunnyvale, CA, USA) from 400 nm to 750 nm. The contents of gold and MB were quantified by inductively coupled plasma mass spectrometry (ICP-MS) and UV-Vis absorbance spectrum analysis, respectively. The loading amount of Au^3+^ was analyzed with ICP-MS using a Varian 820-MS system (Varian, Sydney, Australia). The MB contents in the various groups were quantified with an emission peak at 686 nm (λ_ex_ 665 nm) by fluorescence measurement using a SpectraMAX M5 (Molecular Devices). The temperature elevation of GMDN was evaluated using an infrared thermal camera (FLIR T420; FLIR System Inc., Danderyd, Sweden) upon 808 nm irradiation by a NIR laser (BWT Beijing Ltd., Beijing, China) at 1.5 W power.

### 2.3. Cellular Uptake

Cellular uptake of nanoclusters was determined by optical imaging of the cell pellets, UV-Vis spectra, cellular TEM imaging, and cellular fluorescence imaging. Murine colon carcinoma CT26 cells were seeded to a 24-well plate at a density of 5 × 10^4^ cells per well and incubated for 12 h. The cells were treated with the various formulations at a PD concentration of 40 μg/mL. After 4 h incubation, cells were harvested and centrifuged at 100× *g* for 3 min. The cell pellet color was visualized by optical imaging. The cell pellet was resuspended with 200 μL of 5% glucose solution, and the absorbance of cell suspension was measured at 600 nm using a SpectraMAX M5 (Molecular Devices). The localization of nanoclusters within tumor cells was visualized by TEM (Talos L120C; Thermo Scientific). To prepare the cell samples for TEM imaging, cell pellets were fixed with Karnovsky’s fixative for 2 h at 4 °C and then washed with cold sodium cacodylate buffer (0.05 M). The cell pellets were treated with 1% osmium tetroxide solution, and then subjected to negative staining with 0.5% uranyl acetate. After dehydration by a series ethanol gradient, the cell pellets were transferred to propylene oxide and embedded in Spurr’s resin as previously reported [3]. Thin sections (60 nm) of embedded cell pellets were cut by a microtome and observed by TEM.

### 2.4. Intracellular ROS Generation

The intracellular ROS level of nanocluster-treated and light-irradiated cells was measured by 2′,7′-dichlorodihydrofluorescein diacetate (H_2_DCFDA) staining. CT26 cells were seeded to a 24-well plate at a density of 5 × 10^4^ cells per well and incubated for 12 h. The various formulations were applied to the wells at a PD concentration of 40 μg/mL. After 4 h, cells were washed with phosphate-buffered saline (PBS) and irradiated with a 660-nm light emitting diode (LED; Mikwang Electronics, Busan, Republic of Korea) at an intensity of 8000 mCd for 30 min. To detect intracellular ROS formation, cells were incubated with 10 μM of H_2_DCFDA (Thermo Scientific) in serum-free RPMI at 37 °C for 15 min. After the cells were washed with PBS, and the fluorescence intensity was visualized with a Leica TCS SP8 confocal microscope (Leica Microsystems, Wetzlar, Germany) and quantified by flow cytometry (FACS Lyric; BD Biosciences, San Jose, CA, USA).

### 2.5. In Vitro Photoresponsive Anti-Cancer Efficacy

The in vitro therapeutic effect of GMDN plus dual irradiation was evaluated by cell viability test and live and dead cell staining. CT26 cells were seeded to a 24-well plate at a density of 5 × 10^4^ cells per well and incubated for 12 h. The various formulations were applied to the wells at a PD concentration of 40 μg/mL. After 4 h, cells were washed with phosphate-buffered saline (PBS) and irradiated with a 660-nm LED or 808-nm laser. After 20 h, cell viability was quantified by 3-(4,5-dimethylthizol-2-yl)-2,5-diphenyltetrazolium bromide (MTT) assay. The cells were incubated with MTT (250 μg/mL)-containing culture medium at 37 °C for 2 h and washed with PBS. The intracellular formazan was dissolved in dimethyl sulfoxide and absorbance was measured at 570 nm. Live and dead cell staining (Thermo Scientific) was performed to visualize the cell killing effect by dual irradiation, as previously described [3]. The cells were stained with calcein acetoxymethyl (2 μg/mL) and propidium iodide (3 μg/mL) at 37 °C for 20 min and observed by fluorescence microscopy (Leica DM IL LED; Leica Microsystems). 

### 2.6. Animal Experiments

Five-week-old female BALB/c mice (Raon Bio, Yongin-si, Korea) were used for in vivo experiments. All animal experiments were performed in accordance with the Guidelines for the Care and Use of Laboratory Animals of the Institute of Laboratory Animal Resources. The study protocol (#SNU-190821-5, 21/08/2019, Seoul National University Institutional Animal Care and Use Committee) was approved by the Institutional Review Board for the use of animals at the College of Pharmacy, Seoul National University.

### 2.7. In Vivo Biodistribution

The in vivo distribution of GMDN was evaluated by molecular imaging of the fluorescence of MB. CT26-bearing BALB/c mice were established by subcutaneous inoculation of 1 × 10^6^ CT26 cells. On day 7 after CT26 inoculation, tumor-bearing mice were intravenously injected with free MB or GMDN at an MB dose of 1.14 mg/kg. In vivo fluorescence images of the mice were collected at 1 h, 6 h, and 24 h after the administration of GMDN using an IVIS Spectrum in Vivo Imaging System (PerkinElmer, Waltham, MA, USA).

### 2.8. In Vivo Anticancer Effect

The in vivo therapeutic effect of systemically injected GMDN was tested in CT26 tumor-bearing model mice exposed to dual light irradiation. To establish the tumor model, 6-week-old female BALB/c mice were subcutaneously inoculated with 1 × 10^6^ CT26 tumor cells in the right flank. On day 7 after inoculation, the various formulations were administered intravenously at a PD dose of 5 mg/kg. At 24 h post-injection, the tumors were irradiated with a 660 nm LED (8000 mCd) and an 808 nm laser (1.5 W/cm^2^) for 5 min each. The temperature of the tumor region was monitored during NIR irradiation using an infrared thermal camera (FLIR T420). The volume of tumors was measured by calipers and calculated as previously reported according to the formula: (Length) × (Width)^2^ × 0.5 [4].

### 2.9. Ex Vivo Killing Effect of T Cells after GMDN Treatment

To assessment of adaptive immune response, tumor cell killing effect of T cells was monitored. GMDN-treated and dual light irradiated mice were sacrificed at 7 days after the treatment and T cells were isolated from the spleen by nylon wool fiber column method as previously reported [3]. Isolated T cells were stained with CellTracker Green CMTPX dye (Thermo Scientific) and CT26 tumor cells were labeled by CellTracker Red CMTPX dye (Thermo Scientific). T cells and CT26 cells were co-cultured at a ratio of 100:1 and a real-time video was recorded using an Operetta High-Content Imaging System (PerkinElmer).

### 2.10. Statistical Analysis

Statistical analysis and visualization of experimental data were performed with a two-sided analysis of variance (ANOVA) with Student-Newman-Keuls post-hoc test using GraphPad Prism 7 (GraphPad Software Inc., San Diego, CA, USA). A *p*-value less than 0.05 was considered statistically significant.

## 3. Results

### 3.1. Characterization of GMDN

The GMDN were characterized for their morphological, physicochemical, and photoresponsive characteristics. Elemental mapping showed that Au, phosphorous, and sulfur co-localized in the nanoclusters, indicating the presence of gold nanoclusters, DNA, and MB, respectively (Figure 2B). The average size of GMDN was 51.7 ± 7.7 nm (Figure 2C). GMDN showed a higher zeta-potential (−28.2 ± 0.6 mV) than PD (−48.2 ± 0.3 mV). The amount of encapsulated Au was similar in GMDN and GDN (Figure 2D), at 129.8 ± 11.7 and 105.7 ± 37.3 ng/μg DNA, respectively. The amount of MB in GMDN was 228.0 ± 0.1 ng/ μg DNA, which was not significantly different from that in PD (Figure 2E). The formation of gold clusters was characterized by analysis of the absorbance spectrum (Figure 2F). Whereas PD and MB-PD did not exhibit any significant absorption peak, GMDN revealed remarkable absorbance at 500–700 nm. Due to the loading of Au, the particle had a photothermal effect (Figure 2G). While TDW, PD, and MB-PD did not exhibit any temperature change upon NIR laser irradiation, the temperatures of GDN and GMDN increased from room temperature to 68.6 ± 1.7 and 65.3 ± 2.1 °C, respectively, at 3 min after NIR irradiation.

### 3.2. Cellular Uptake of GMDN

**Figure 3:**
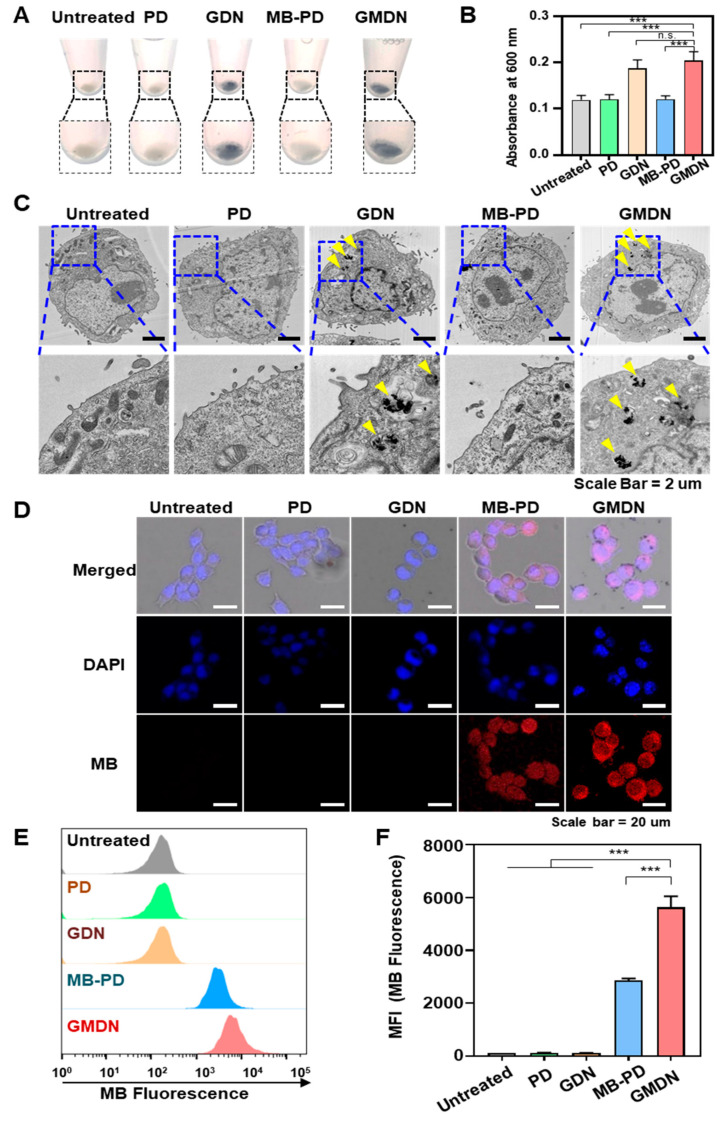
Cellular uptake of nanoclusters. (**A**) Cell pellets were observed after cells were treated with PD, GDN, MB-PD, or GMDN. (**B**) Absorbances of cell suspensions were measured at 600 nm. (**C**) Cellular internalization of gold nanoclusters was observed by TEM imaging. (**D**) Cells were treated with the various formulations and fluorescence was observed by confocal microscopy. (**E**,**F**) Cellular uptake of nanoclusters was measured by flow cytometry (**E**) and analysis of average fluorescence intensity (**F**) (*** *p* < 0.001).

The cellular uptake of the gold nanoclusters was observed based on cell color, cellular TEM imaging, and fluorescence microscopy. First, the cellular uptake of GMDN was visualized by the color of the cell pellet (Figure 3A). The pellets of GDN- and GMDN-treated cells were much darker than those of the other groups, and GDN- and GMDN-treated cells had much higher absorbances than the other groups (Figure 3B). Gold nanoclusters were observed in the cytoplasm of GDN- and GMDN-treated cells (Figure 3C). The cellular uptake of GMDN was also evaluated by assessing the fluorescence of MB. PD- and GDN-treated cells did not exhibit any fluorescence, whereas the MB-PD- and GMDN-treated groups showed strong fluorescence, indicating cellular uptake of MB. Cells treated with GMDN showed a higher uptake rate compared to those treated with MB-PD, as assessed by examining the fluorescence of methylene blue by FACS analysis (Figure 3E). The nanosized clusters of GMDN had 2.0-fold higher fluorescence than the microsized structures of MB-PD (Figure 3F).

### 3.3. In Vitro Phototherapeutic Effects of GMDN

**Figure 4:**
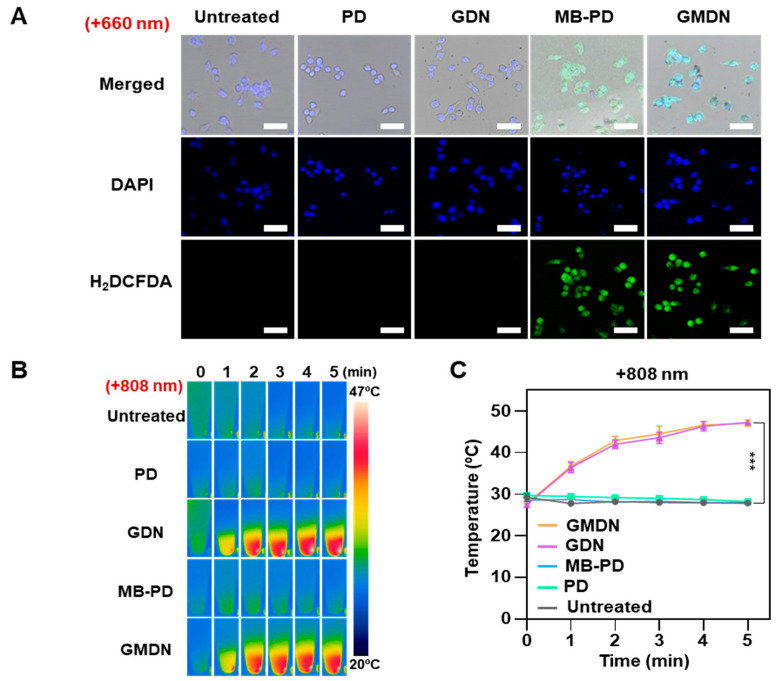

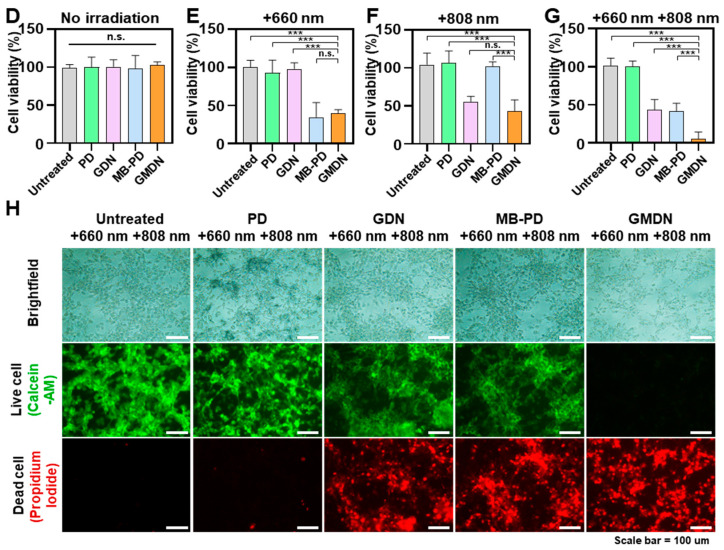
In vitro photodynamic and photothermal effects of GMDN. Various formulations were applied to CT26 cells for 4 h, followed by 660-nm or 808-nm irradiation. (**A**) ROS generation in the cells was evaluated by H2DCFDA detection upon 660-nm irradiation. (**B**) Upon 808-nm NIR laser irradiation, heat generation was observed by thermo-imaging. (**C**) The temperature was monitored during irradiation. (**D**–**G**) Anti-cancer efficacy was evaluated by measuring cell viability under no irradiation (**D**), 660-nm irradiation (**E**), 808-nm irradiation (**F**) and dual light irradiation (**G**). (**H**) The anti-cancer efficacy was visualized by live and dead cell imaging (n.s., not significant; *** *p* < 0.001).

The dual light responsiveness of GMDN resulted in ROS generation and temperature increase, which was able to kill cancer cells. Upon 660-nm irradiation, ROS generation was not detected in control groups treated with PD or GDN (Figure 4A). However, MB-PD- and GMDN-treated CT26 cells showed robust ROS generation. Photothermal efficacy was observed only in GDN- and GMDN-treated cells (Figure 4B,C). Upon NIR laser irradiation, negligible temperature increases were observed in PD- and MB-PD-treated cells, which lacked Au in the formulation (Figure 4B). In contrast, significant temperature increases upon NIR irradiation were confirmed in GDN- and GMDN-treated cells, which exhibited increases of up to 47.2 ± 0.5 and 47.1 ± 0.8 °C, respectively (Figure 4C). The ROS-mediated photodynamic effect and heat-mediated photothermal effect led to synergistic anti-cancer efficacy. Whereas cell viability was not affected by any of the tested formulations in the absence of irradiation (Figure 4D), ROS generation upon 660-nm irradiation significantly reduced the cell viability of the GMDN group to 40.4% (Figure 4E). Heat generation upon 808-nm NIR irradiation also induced significant cell death, with the GMDN-treated group showing 43.1% cell viability (Figure 4F). When both lights were applied to the cells, a synergistic anti-cancer effect was confirmed (Figure 4G). The GDN and MB-PD groups showed comparable cell viabilities above 40% upon dual light irradiation, whereas GMDN-treated cells showed significantly decreased cell viability under dual light irradiation, down to 5.4 ± 9.1% (Figure 4G). The results of live and dead cell staining supported this synergistic anti-cancer efficacy by showing significantly lower levels of living cells and higher levels of dead cells when the dual light was applied to GMDN-treated cells compared to the GDN- or MB-PD-treated groups (Figure 4H).

### 3.4. In Vivo Anti-Tumor Efficacy of GMDN

**Figure 5:**
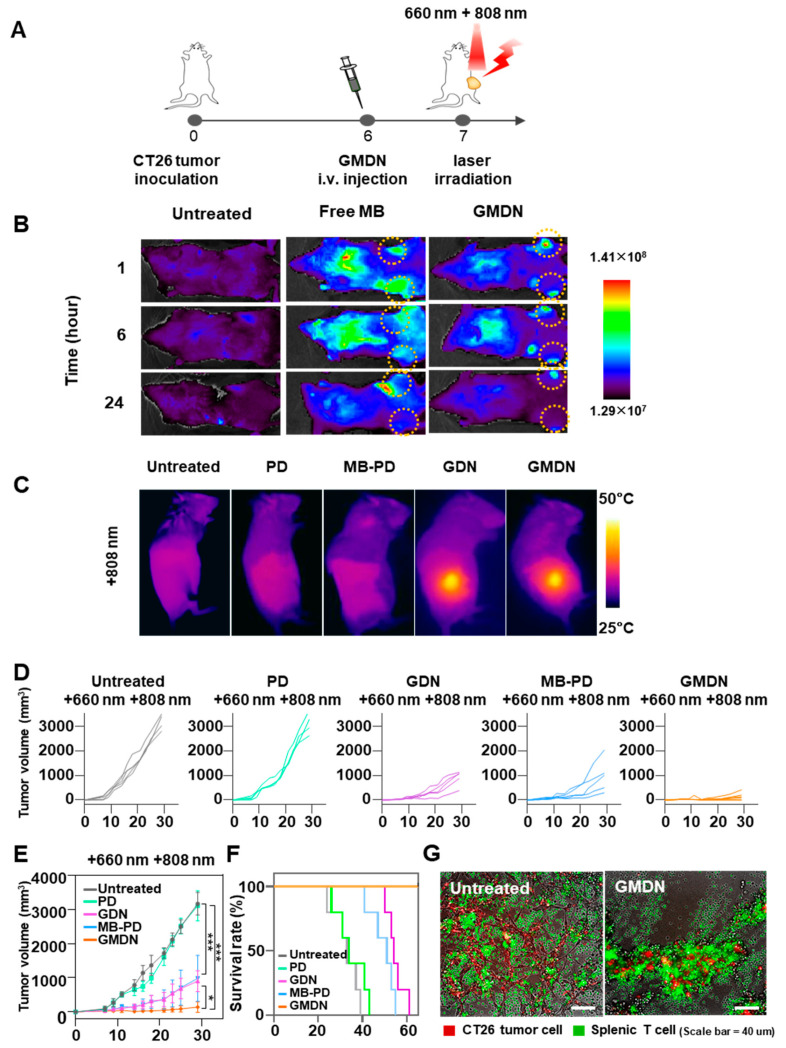
In vivo synergistic dual phototherapy of GMDN. The in vivo anti-tumor efficacy of GMDN was evaluated in CT26 tumor-bearing mice after light irradiation. (**A**) After CT26 tumor inoculation, GMDN were intravenously injected, followed by dual irradiation. (**B**) In vivo biodistribution of GMDN was evaluated by detecting fluorescence intensity from MB. (**C**) In vivo photothermal effect was confirmed by temperature measurement at the tumor site upon NIR irradiation. (**D**,**E**) In vivo anti-tumor efficacy was evaluated by tumor volume measurement up to 30 days after first tumor inoculation. (**F**) Survival was monitored for 65 days. (**G**) Anti-tumor efficacy was evaluated in a tumor cell co-culture model involving splenic T cells from treated or untreated mice (* *p* < 0.05; *** *p* < 0.001).

The in vivo distribution and synergistic dual phototherapeutic effects of GMDN were confirmed in vivo (Figure 5A). Each formulation was intravenously administered to CT26 tumor-bearing mice. Our results revealed that free MB was distributed throughout the whole body (Figure 5B). However, the fluorescence signal was significantly increased at the tumor site of the GMDN-treated group, and lower distribution was seen in other organs (e.g., liver) under this treatment. Moreover, the accumulation of GMDN resulted in a temperature increase from room temperature to 47.1 ± 1.7 °C upon 808-nm NIR irradiation at the tumor site (Figure 5C). When dual light was applied to the tumor, slight suppression of tumor growth was observed in the GDN- and MB-PD-injected groups, which showed average tumor volumes of 902.6 ± 306.9 and 981.3 ± 671.9 mm^3^, respectively, while the average tumor volume of the untreated group was 3169.1 ± 328.6 mm^3^ (Figure 5D,E). However, dual light irradiation of the GMDN-treated group significantly inhibited tumor growth even further, yielding an average tumor volume of 140.0 ± 156.1 mm^3^. As a result, GMDN improved the survival of the mice (Figure 5F). While no mouse survived to day 61 post tumor inoculation in any other group, GMDN-treated mice showed 100% survival up to day 65 (Figure 5F). When splenic T cells were extracted from the surviving mice and co-cultured with tumor cells, a more robust anti-cancer response was observed compared to that obtained with naïve splenic T cells from untreated mice (Figure 5G).

## 4. Discussion

Here, we developed dual light-responsive nanocluster GMDN, which can exert a dual phototherapeutic effect for cancer phototherapy. Our use of a long DNA strand produced from RCA as a backbone for intercalating MB and clustering gold ions conferred multi-functionality to the nanoclusters as a theranostic formulation.

We used RCA to generate a repeated-sequence long PD strand that was then used as a scaffold for therapeutic cargo loading. As a natural biopolymer, the DNA nanostructure has great potential in nanotechnology [21]. For example, the fabrication and construction of a DNA-based structure can be easily controlled due to its intrinsic intermolecular interactions. DNA can form duplex, hairpin, loop, and/or G-quadruplex structures, which can endow the nanostructure with unique functions. The ease of sequence design can also give additional functionality. Most of all, DNA is a biocompatible and safe material that is suitable for clinical application.

The robust nanocluster formation by PD, MB, and gold was confirmed by elemental analysis. MB is a ROS-generating photoresponsive dye and DNA-intercalating agent [15]. By binding to double-stranded DNA with high affinity, the MB and PD formed a stable MB-PD complex. When gold ions were reduced to gold clusters on the PD backbone, the bulky PD structure was compacted to nano-size and MB-entrapping gold nanoclusters were formed.

The resulting GMDN nanoparticles could respond to dual light for photothermal and photodynamic therapy, respectively. When the GMDN were applied to cancer cells, the particles were efficiently internalized, whereas this was not true for the micro-sized bulky nanostructures used for comparison (Figure 3F). The enhanced cellular uptake due to nanocluster formation was associated with a synergistic anti-cancer effect. Our in vitro studies revealed that while photothermal therapy or photodynamic therapy alone could not induce significant anti-cancer efficacy, the simultaneous treatment of dual phototherapy resulted in a dramatic anti-cancer killing effect (Figure 4G,H). The use of dual lights has been reported to exert synergistic anticancer efficacy compared to single light irradiation [22,23,24]. The synergistic effects can be explained by several factors. First, the combination of dual lights has been shown to overcome the limited penetration depth of red light. Secondly, the hyperthermia condition by photothermal therapy at 808 nm has been observed to increase the uptake of photosensitizers by tumor cells [23]. Thirdly, the singlet oxygen generated by photosensitizers at 660 nm is known to attack heat-shock proteins, enhancing the photothermal effect by 808 nm [25].

GMDN can enable the imaging of tumor tissues, as MB has been used in clinical settings as a cancer diagnostic dye. When GMDN were administered intravenously, significant accumulation in tumor tissue was observed. Since nanoparticles are intrinsically able to accumulate in tumor tissues through the enhanced retention and permeability effect, a nano-formulation that includes an imaging agent can aid in tumor diagnosis [26]. The biodistribution results obtained in this study suggest that GMDN showed improved tumor imaging with higher selectivity, as compared to the same amount of free MB. 

For translation of this study to the colon cancer patients, optical fibers need to be used as a light source to irradiate the deep lesion of the patients [27]. A recent study reported that a 100 μm-diameter optical fiber could provide photothermal ablation of colorectal cancer metastasized to the liver [28]. In another study, an optical fiber with a 20-mm cylindrical diffuser has been used for photodynamic therapy of cancer patients [29]. Molecular imaging data obtained through GMDN might be useful for the treatment of image-guided phototherapy, which requires access to the correct lesion using optical fibers. 

GMDN also showed a synergistic anti-cancer effect in an animal model. When tumor growth was monitored for 30 days after photo-treatment, photothermal therapy or photodynamic therapy alone showed limited therapeutic efficacy with continued tumor growth. However, complete tumor ablation was observed at day 65 after dual photothermal and photodynamic therapy, and no mortality was recorded during this period.

## 5. Conclusions

Although this study applied dual phototherapy with GMDN, the scope of application can be broadened. Various anti-cancer strategies could be applied using GMDN, such as by inserting a therapeutic sequence or loading a functional oligonucleotide to the platform DNA backbone. In addition to MB, other various types of DNA-intercalating agent could also be incorporated to diversify the function of the nanoparticle. Our results suggest that GMDN could function as a photoresponsive theranostic for cancer treatment. In particular, the phototherapy-induced immunogenic cell death leads us to test whether the acquired adaptive immunity by GMDN can prevent metastasis and recurrence of cancer. In the near future, the safety profiles, and efficacy studies in various tumor-bearing animal models needs to be done. The safety profiles obtained in repeated dosing would be critical to proceed to the clinical trials in the future.

## References

[1] Li F., Qin Y., Lee J., Liao H., Wang N., Davis T.P., Qiao R., Ling D. (2020). Stimuli-responsive nano-assemblies for remotely controlled drug delivery. J. Control. Release.

[2] Kim D., Byun J., Park J., Lee Y., Shim G., Oh Y.-K. (2020). Biomimetic polymeric nanoparticle-based photodynamic immunotherapy and protection against tumor rechallenge. Biomater. Sci..

[3] Le Q.-V., Suh J., Choi J.J., Park G.T., Lee J.W., Shim G., Oh Y.-K. (2019). In Situ Nanoadjuvant-Assembled Tumor Vaccine for Preventing Long-Term Recurrence. ACS Nano.

[4] Shim G., Ko S., Park J.Y., Suh J.H., Le Q.-V., Kim D., Kim Y.B., Im G.H., Kim H.N., Choe Y.S. (2020). Tannic acid-functionalized boron nitride nanosheets for theranostics. J. Control. Release.

[5] Cho M.H., Li Y., Lo P.-C., Lee H., Choi Y. (2020). Fucoidan-Based Theranostic Nanogel for Enhancing Imaging and Photodynamic Therapy of Cancer. Nano-Micro Lett..

[6] Chen Y., Gao Y., Chen Y., Liu L., Mo A., Peng Q. (2020). Nanomaterials-based photothermal therapy and its potentials in antibacterial treatment. J. Control. Release.

[7] Zhao X., Yang C.-X., Chen L.-G., Yan X.-P. (2017). Dual-stimuli responsive and reversibly activatable theranostic nanoprobe for precision tumor-targeting and fluorescence-guided photothermal therapy. Nat. Commun..

[8] Shim G., Le Q.-V., Suh J., Choi S., Kim G., Choi H.-G., Kim Y.B., MacGregor R.B., Oh Y.-K. (2019). Sequential activation of anticancer therapy triggered by tumor microenvironment-selective imaging. J. Control. Release.

[9] Yang H., Le Q.-V., Shim G., Oh Y.-K., Shin Y.K. (2020). Molecular engineering of antibodies for site-specific conjugation to lipid polydopamine hybrid nanoparticles. Acta Pharm. Sin. B.

[10] Li X., Lovell J.F., Yoon J., Chen X. (2020). Clinical development and potential of photothermal and photodynamic therapies for cancer. Nat. Rev. Clin. Oncol..

[11] Choi K., Riviere J.E., Monteiro-Riviere N.A. (2017). Protein corona modulation of hepatocyte uptake and molecular mechanisms of gold nanoparticle toxicity. Nanotoxicology.

[12] Fadeel B., Bussy C., Merino S., Fernandez-Pacheco E.V., Laurent C., Mouchet F., Evariste L., Gauthier L., Koivisto A.J., Vogel U. (2018). Safety Assessment of Graphene-Based Materials: Focus on Human Health and the Environment. ACS Nano.

[13] Huang Y.-Y., Wintner A., Seed P.C., Brauns T., Gelfand J.A., Hamblin M.R. (2018). Antimicrobial photodynamic therapy mediated by methylene blue and potassium iodide to treat urinary tract infection in a female rat model. Sci. Rep..

[14] Dos Santos A.F., Terra L.F., Wailemann R.A.M., Oliveira T.C., Gomes V.D.M., Mineiro M.F., Meotti F.C., Bruni-Cardoso A., Baptista M.S., Labriola L. (2017). Methylene blue photodynamic therapy induces selective and massive cell death in human breast cancer cells. BMC Cancer.

[15] Jin H., Kim M.G., Ko S.B., Kim D.-H., Lee B.-J., MacGregor J.R.B., Shim G., Oh Y.-K. (2018). Stemmed DNA nanostructure for the selective delivery of therapeutics. Nanoscale.

[16] Shim G., Park J., Kim M.-G., Yang G., Lee Y., Oh Y.-K. (2020). Noncovalent tethering of nucleic acid aptamer on DNA nanostructure for targeted photo/chemo/gene therapies. Nanomedicine.

[17] Ali M.R.K., Wu Y., El-Sayed M.A. (2019). Gold-Nanoparticle-Assisted Plasmonic Photothermal Therapy Advances Toward Clinical Application. J. Phys. Chem. C.

[18] Siddique S., Chow J.C.L. (2020). Application of Nanomaterials in Biomedical Imaging and Cancer Therapy. Nanomaterials.

[19] Siddique S., Chow J.C.L. (2020). Gold Nanoparticles for Drug Delivery and Cancer Therapy. Appl. Sci..

[20] Rastinehad A.R., Anastos H., Wajswol E., Winoker J.S., Sfakianos J.P., Doppalapudi S.K., Carrick M.R., Knauer C.J., Taouli B., Lewis S.C. (2019). Gold nanoshell-localized photothermal ablation of prostate tumors in a clinical pilot device study. Proc. Natl. Acad. Sci. USA.

[21] Seeman N. (1999). DNA Nanotechnology. Nat. Biotechnol..

[22] Shim G., Kim M.-G., Jin H., Kim J., Oh Y.-K. (2017). Claudin 4-targeted nanographene phototherapy using a Clostridium perfringens enterotoxin peptide-photosensitizer conjugate. Acta Pharmacol. Sin..

[23] Liu X., Su H., Shi W., Liu Y., Sun Y., Ge D. (2018). Functionalized poly(pyrrole-3-carboxylic acid) nanoneedles for dual-imaging guided PDT/PTT combination therapy. Biomaterials.

[24] Li W., Yang J., Luo L., Jiang M., Qin B., Yin H., Zhu C., Yuan X., Zhang J., Luo Z. (2019). Targeting photodynamic and photothermal therapy to the endoplasmic reticulum enhances immunogenic cancer cell death. Nat. Commun..

[25] Cheng Q., Li Z.-H., Sun Y.-X., Zhang X. (2019). Controlled synthesis of a core-shell nanohybrid for effective multimodal image-guided combined photothermal/photodynamic therapy of tumors. NPG Asia Mater..

[26] Fang J., Islam W., Maeda H. (2020). Exploiting the dynamics of the EPR effect and strategies to improve the therapeutic effects of nanomedicines by using EPR effect enhancers. Adv. Drug Deliv. Rev..

[27] Khot M.I., Andrew H., Svavarsdottir H.S., Armstrong G., Quyna A.J., Jaynea D.G. (2019). A Review on the Scope of Photothermal Therapy–Based Nanomedicines in Preclinical Models of Colorectal Cancer. Clin. Color. Cancer.

[28] Parchur A.K., Sharma G., Jagtap J.M., Gogineni V.R., LaViolette P.S., Flister M.J., White S.B., Joshi A. (2018). Vascular Interventional Radiology-Guided Photothermal Therapy of Colorectal Cancer Liver Metastasis with Theranostic Gold Nanorods. ACS Nano.

[29] Shafirstein G., Battoo A., Harris K., Baumann H., Gollnick S.O., Lindenmann J., Nwogu C.E. (2016). Photodynamic therapy of non–small cell lung cancer. Narrative review and future directions. Ann. Am. Thorac. Soc..

